# The mechanism of electroacupuncture for depression on basic research: a systematic review

**DOI:** 10.1186/s13020-020-00421-y

**Published:** 2021-01-13

**Authors:** Xuke Han, Yang Gao, Xuan Yin, Zhangjin Zhang, Lixing Lao, Qiu Chen, Shifen Xu

**Affiliations:** 1 Hospital of Chengdu University of Traditional Chinese Medicine, Chengdu, 610072 Sichuan China; 2 Shanghai Municipal Hospital of Traditional Chinese Medicine, Shanghai University of Traditional Chinese Medicine, Shanghai, 200071 China; 3 School of Chinese Medicine, The University of Hong Kong, Pokfulam, Hong Kong SAR, China; 4 Virginia University of Integrative Medicine, Fairfax, Virginia 22031 USA

**Keywords:** Electroacupuncture (EA), Depression, Mechanism, Animal research, Review

## Abstract

**Background:**

Electroacupuncture (EA) is generally accepted as a safe and harmless treatment option for alleviating depression. However, there are several challenges related to the use of EA. Although EA has been shown to be effective in treating depression, the molecular mechanism is unclear.

**Objective:**

To reveal the therapeutic effect of EA and its possible mechanism in the treatment of depression.

**Search strategy:**

We performed a systematic search according to PRISMA guidelines. We electronically searched PubMed, Web of Science (WOS), the China National Knowledge Infrastructure (CNKI), Wanfang Data Information Site and the VIP information database for animal studies in English published from the inception of these databases to December 31, 2019.

**Inclusion criteria:**

Electronic searches of PubMed, WOS, the CNKI, Wanfang and the VIP database were conducted using the following search terms: (depression OR depressive disorder OR antidepressive), (rat OR mouse) AND (acupuncture OR EA).

**Data extraction and analysis:**

The data were extracted primarily by one author, and a follow-up review was conducted by the other authors.

**Results:**

Twenty-eight articles met the inclusion criteria. The most commonly used method for inducing depression in animal models was 21 days of chronic unpredictable mild stress. For the depression model, the most commonly selected EA frequency was 2 Hz. Among the 28 selected studies, 11 studies observed depression-related behaviors and used them as indicators of EA efficacy. The other 17 studies focused on mechanisms and assessed the indexes that exhibited abnormalities that were known to result from depression and then returned to a normal range after EA treatment. Treatment of depression by EA involves multiple therapeutic mechanisms, including inhibition of HPA axis hyperactivity and inflammation, regulation of neuropeptides and neurotransmitters, modulation of the expression of particular genes, restoration of hippocampal synaptic plasticity, increased expression of BDNF, and regulation of several signaling pathways.

**Conclusions:**

This review reveals that the mechanisms underlying the effect of acupuncture involve multiple pathways and targets, suggesting that acupuncture is a wholistic treatment for people rather than for diseases. Our findings also explain why acupuncture can treat various disorders in addition to depression.

## Introduction

Depression is a common disease characterized by emotional dysfunction, and the main symptom is a significant and enduring low mood [1]. According to the World Health Organization (WHO), by 2020, depression will be the 2nd most damaging public health threat worldwide [2]. Depression not only affects the nervous system but can also induce lesions in multiple systems [3]. Moreover, the incidence of depression is increasing, while the cure rate is decreasing [4]. The clinical application of Western medicine approaches for the treatment of depression is limited due to their side effects. Some articles have reported the occurrence of adverse effects such as sexual dysfunction, gastrointestinal reactions, anxiety symptoms, sleepiness and suicidal tendencies following the use of Western antidepressants [5–9]. Acupuncture is a complimentary treatment option that involves inserting stainless steel needles into the skin at specified points called acupoints to physically stimulate the body. Traditional Chinese acupuncture treatment has been shown to have an effect on nervous system diseases [10, 11]. Electroacupuncture (EA), which is based on traditional Chinese medicine acupuncture, uses an electrical device connected to a needle to send electrical currents to the acupoint and thereby help stimulate it. Because it is modifiable, safe and does not pose a risk for addiction, EA provides advantages for the treatment of depression [10–12]. As a vital therapeutic modality in complementary and alternative medicine, EA treatment has been verified to be safe and efficacious in patients with depression in previous clinical studies  [13, 14]. Numerous studies have shown that EA is well tolerated by patients and as safe and effective as routine care. EA has displayed potent antidepressant-like effects in many clinical studies [15]. In an 8-week controlled clinical trial, EA as a therapy for depression appeared to result in greater symptom improvement with respect to anxiety and feelings of despair than selective serotonin reuptake inhibitor (SSRI) treatment [16]. Another clinical study reported that EA and fluoxetine have similar curative effects on depression. EA has a faster onset of action and higher response rate and induces greater improvements than fluoxetine [17]. Additionally, Duan et al. reported that EA at GV20 can modulate abnormal aberrant amygdala networks in depressed patients, providing further imaging evidence to support the modulatory mechanisms of EA in depression [14].

Although EA has been confirmed to alter psychological function, the mechanism of EA in depression has not yet been clarified. Considering the important effects of EA intervention on emotional systems and its implication in depression, the present study focuses on studies that use EA to treat depression in animals. The available literature on the mechanism of EA therapy in depression in animals in recent years is reviewed. Our goal is to better illuminate the therapeutic effect of EA treatment and its possible mechanism in the treatment of depression.

## Methods

### Search strategies

We searched the PubMed, Web of Science (WOS), the China National Knowledge Infrastructure (CNKI), Wanfang Data Information Site and the VIP information database using the terms depression OR depressive disorder OR antidepressive AND rat OR mouse AND acupuncture OR EA. Our search was limited to animal research published in English from the inception of these databases to December 31, 2019.

We selected articles that described studies of the effects of EA on depression in animal models. Clinical studies of depression were excluded, as were review articles based on the literature and studies of animal models of other types of mental illness, such as anxiety, neurosis and schizophrenia. We also excluded studies that did not focus on rat or mouse models and that did not use an EA-related treatment approach. The full texts of the articles meeting the inclusion criteria were obtained and read carefully.

## Data extraction

The study design data were extracted and classified using a predefined data extraction form that determined the animal used (sex, species, and strain), depression animal model type (type of stress), type of intervention (the acupoints used and method of stimulation) and outcome measures (depression-related behavioral tests, appearance characteristics, electrophysiological indicators, histologic examination and biochemical measurements). The data were extracted primarily by one author, and a follow-up review was conducted by the other authors.

## Results

**Fig. 1:**
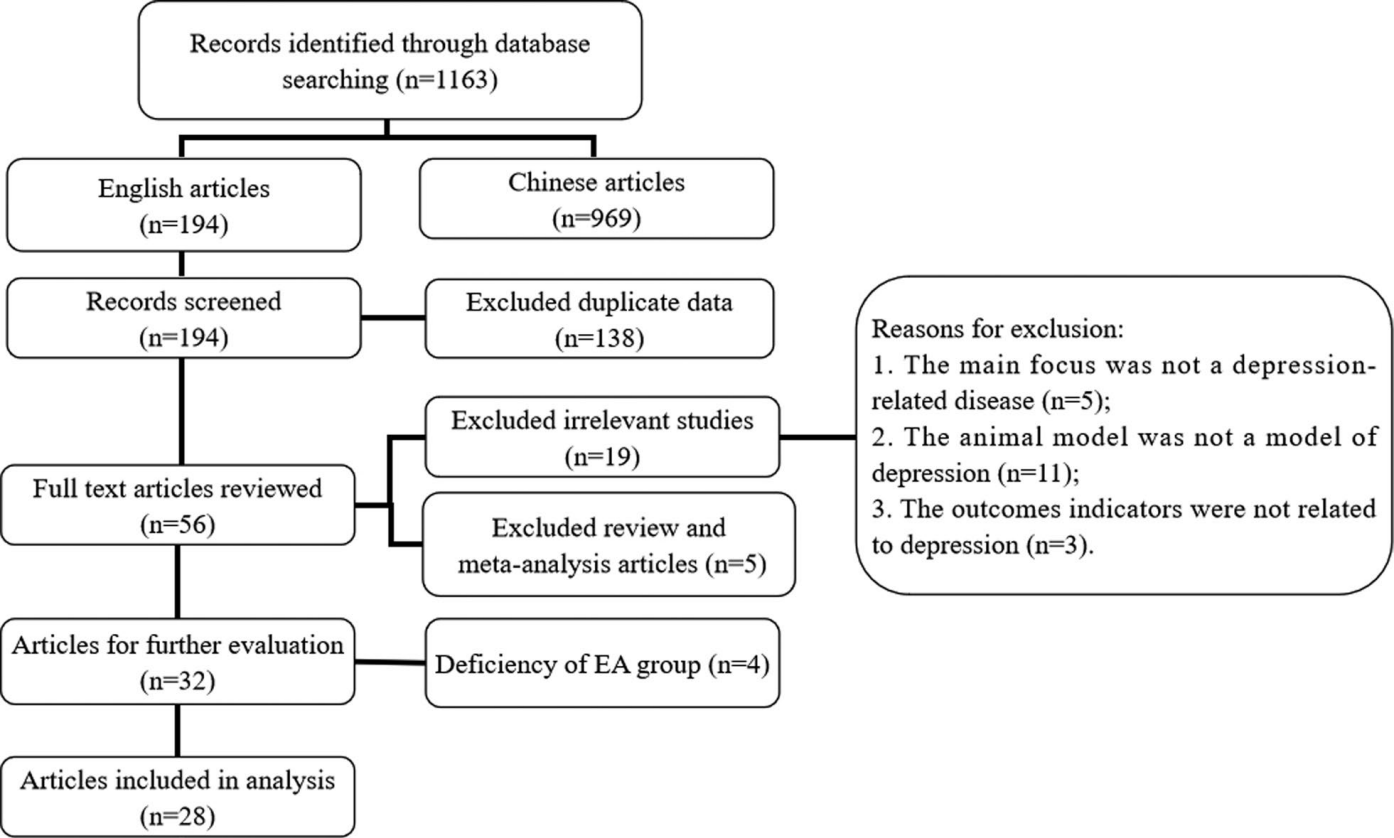
Flow diagram of the study selection process

We retrieved 1163 articles and read the titles and abstracts. There were 969 Chinese articles and 194 English articles. Of these 194 English articles, 138 articles were replication studies, and 5 articles were reviews or meta-analyses of the literature. Nineteen articles were irrelevant to our research: (1) The main focus was not a depression-related disease; (2) The animal model was not a model of depression; or (3) The outcomes indicators were not related to depression. The remaining 32 articles were reviewed, and 4 were excluded because they did not use EA therapy (only traditional acupuncture). Therefore, the remaining 28 articles were included in the review (Fig. 1).

According to the data analysis, most animal experiments utilized the GV29/EX-HN3 (Yintang) and GV20/DU20 (Baihui) acupoints to treat depression. The anode was inserted into GV29, and the cathode was inserted into GV20. It can be inferred that GV20 and GV29 are considered the best acupoint modules for depression. In most studies, the EA frequency was held constant at 2 Hz, but the pulse width and intensity and needle retention time varied according to the specific design of each experiment.

We analyzed the animal models of depression used in the 28 studies (Table 1). Of these studies, 3 used Wistar Kyoto (WKY) rats as a well-studied model of depression, 2 used the chronic mild stress (CMS)-induced depression rat model and 15 used the chronic unpredictable mild stress (CUMS)-induced depression rat model. The remaining 4 articles used a chronic restraint stress (CRS)-induced depression rat model, a pain-depression rat model, a poststroke depression (PSD) rat model and a model of depression induced by injection of 6-hydroxydopamine into the right medial forebrain bundle (MFB). In short, the most commonly used depression models were the CUMS model and WKY rats. During the CUMS procedure, animals are exposed to a variable sequence of mild unpredictable chronic restraint stress, preventing habituation. Rats are randomly exposed to one of the following stressors: food and water deprivation, cage tilt and rotation, lights on/off, cold stress, swimming stress and crowding or isolation [18]. The WKY rat strain has been characterized as a genetic model of depression. WKY rats show various depressive symptoms that mimic those observed in humans, such as exaggerated immobility in the FS and low social activity [19]. This strain also shows resistance to the antidepressant effects of selective serotonin reuptake inhibitors, suggesting that it may provide insight into the mechanisms that confer resistance to antidepressant treatment.

**Table 1:** Models of depression

Animal	Model type	Modeling time	Microbiology level	Sex	Strain	References
Rat	WKY	-	SPF	Male	Wistar	[20–22]
	CUMS	56 days 28 days	SPF SPF	Male Male	Sprague–Dawley Wistar Wistar	[23] [24] [25]
	CUS	70 days 49 days 28 days	SPF SPF SPF	Male Male Male	Sprague–Dawley Sprague–Dawley Sprague–Dawley	[26] [27] [28]
	CUMS	21 days	SPF CL	Male Male	Sprague–Dawley Wistar Sprague–Dawley	[29–33] [34–37] [38]
	CUMS	14 days	SPF	Male	Sprague–Dawley	[39]
	CRS	21 days 28 days	SPF SPF	Male Male	Sprague–Dawley Sprague–Dawley	[40] [41]
	CMS	28 days 77 days	SPF SPF	Male Male	Sprague–Dawley Sprague–Dawley	[42] [43]
	Pain-depression dyad	3 days	SPF	Male	Sprague–Dawley	[44]
	PSD	3 days	SPF	Male	Sprague–Dawley	[45]
	6-OHDA-lesioned	-	SPF	Male	Sprague–Dawley	[46]
	MS	21 days	SPF	Male	Wistar	[47]

*WKY* Wistar Kyoto, *CUMS* chronic unpredictable mild stress, *CRS* chronic restraint stress, *6-OHDA *6-hydroxydopamine, *PSD* poststroke depression, *MS* maternal separation, *SPF* specified pathogen free, *CL* clean animal

All 28 studies investigated the effects of EA on depression (Table 2). Of these studies, 11 assessed the effect of EA on depression-related behavior, including performance in the forced swim test (FST), open-field test (OFT), Morris water maze (MWM) test, and sucrose preference test (SPT), and body weight [20, 21, 23, 24, 27, 29, 34, 42–45]. Four other studies assessed changes in neuropeptides and neurotransmitters, including the protein and mRNA expression of galanin (Gal) and tryptophan hydroxylase (TPH), and serotonin (5-HT) levels [21, 25, 30, 45]. Three additional studies assessed changes in hippocampal synaptic plasticity [21, 22, 35]. Moreover, 2 studies assessed changes in proinflammatory cytokine levels [26, 40], and 2 studies assessed changes in the hypothalamic-pituitary-adrenal (HPA) axis [26, 40]. Three other studies assessed changes in BDNF levels [27, 36, 46], while 6 studies evaluated the effects of EA on signaling pathways, including the AC-cAMP-PKA, Shh, extracellular regulated protein kinase (ERK), and mitogen-activated protein kinase (MAPK) signaling pathways, and dopaminergic synapses [28, 31, 32, 39, 41, 45]. The final 2 studies investigated changes in the expression of multiple genes after EA therapy in depressed rats [37, 47].

**Table 2:** Interventions and outcomes

Reference	Intervention	Number of animals	Acupoint	Stimulation	Control	Number of animals	Days of EA treatment^a^	Outcome measure	Assessment days	Result	Significance
[20]-1	EA	7	GV14 GV20	2 Hz, 0.3 ms, < 3 mA, 15 min	Sham EA (GV14 GV20, no stimulation)	6	Days 8–29	FST OFT MWM	3 weeks after the initial EA treatment	Decreased Increased	*p< 0.05*
[34]-1	EA	8	ST36 CV4	100 Hz 3 s/ 2 Hz 3 s, 1 mA, 20 min	CUMS only No intervention	8	Days 21–35	FST OFT	24 h after the last EA treatment	Decreased Increased	*p < 0.01*
[34]-2	EA	8	ST36 CV4	100 Hz 3 s/ 2 Hz 3 s, 1 mA, 20 min	CUMS only No intervention	8	Days 21–35	CRH mRNA CORT ACTH	Afterbehavioral tests	Decreased	*p< 0.01* *p< 0.05* *p< 0.05*
[24]-1	EA	15	GV20 GV29	2 Hz, 1 mA, 20 min	CUMS only No intervention	15	Days 4-end of the study	Body weight OFT	0,28	Increased	*p*< 0.01
[23]-1	EA	8	GV20 GV29	2 Hz, 1 mA, 20 min	Sham EA (GV20 GV29 no stimulation)	8	Days 31–59	Body weight OFT SPT	3, 31, 33, 59	Increased	*P* > 0.05 *p*< 0.05
[27]-1	EA + citalopram 5 mg/kg	8	GV20 EX-HN3	2 Hz/100 Hz, < 3 mA 0.2 ms,30 min	CUMS only No intervention/ Citalopram (10 mg/kg)	8	Days 28–49	FST OFT SPT	7,14,21,28, 35,42,49	Decreased Increased	*p*< 0.01
[27]-2	EA + citalopram 5 mg/kg	8	GV20 EX-HN3	2 Hz/100 Hz, < 3 mA 0.2 ms,30 min	CUMS only No intervention/ Citalopram (10 mg/kg)	8	Days 28–49	mBDNF proBDNF TrkB	After behavioral tests	Increased	*p*< 0.001 *p*< 0.01 *p*< 0.001
[44]-1	EA	10	ST36 SP6	2, 15, 100 Hz, 2/100 Hz, 0.2 ms,15 min	LI11 TE5	10	Days 4–5	EZM OFT	30 min after EA treatment	Increased	*p*< 0.05
[29]-1	EA	12	ACR	2 Hz, 1 mA, 20 min	Ear-tip CUMS only No intervention	8 12	Days 22–36	HR BP OFT	0,22,36,50	Decreased	*p*< 0.01
[21]-1	EA	8	GV20 EX-HN3	2 Hz, 0.2 ms, 0.1-4 mA, 15 min	Sham EA (GV20, EX-HN 3 no stimulation)	8	Days 1–21	FST OFT SPT	12 h after EA treatment	Decreased Increased	*P < 0.05* *P < 0.01*
[21]-2	EA	8	GV20 EX-HN3	2 Hz, 0.2 ms, 0.1–4 mA, 15 min	Sham EA (GV20, EX-HN 3 no stimulation)	8	Days 1–21	LTP 5-HTT 5-HT1A	After behavioral tests	Increased Decreased	*p< 0.01*
[45]-1	EA	8	GV20 DU24	2 Hz, 30 min	Poststroke depression only No intervention	8	Days 3–31	GSH IL1β TNFα, IL6	Day 31 after EA treatment	Increased Decreased Decreased Decreased	*P < 0.05* *-* *p < 0.001* *p < 0.05*
[45]-2	EA	8	GV20 DU24	2 Hz, 30 min	Poststroke depression only No intervention	8	Days 3–31	Smo, Shh, Gli1, Ptch1 5-HT	Day 31 after EA treatment	Increased	*P < 0.05* *P < 0.01* *P < 0.01*
[42]-1	EA + chlorimi-pramine (2.5 mg/kg)	10	DU20 EX17	60 Hz/5 s–4 Hz/2.5 s, 40 min	CMS only/ chlorimipramine (2.5 mg/kg)	10	Weeks 5–11	SPT FST	After 11 weeks	Increased Decreased	*P < 0.05*
[42]-2	EA + chlorimi-pramine (2.5 mg/kg)	10	DU20 EX17	60 Hz/5 s–4 Hz/2.5 s 40 min	chlorimipramine (5 mg/kg)	10	Weeks 5–11	SPT FST	After 11 weeks	Decreased	*-* *P < 0.05*
[43]-1	EA	8	DU20 EX17	60 Hz/5 s–4 Hz/2.5 s, 40 min	CMS only	8	Weeks 5–11	SPT FST Body weight	After 11 weeks	Increased Decreased –	*P < 0.05* *P < 0.01* *-*
[43]-2	EA + chlorimi-pramine (2.5 mg/kg)	8	DU20 EX17	60 Hz/5 s–4 Hz/2.5s s, 40 min	Chlorimipramine (2.5 mg/kg) Chlorimipramine (5 mg/kg)	8	Weeks 5–11	SPT FST Body weight	After 11 weeks	Increased Decreased –	*P < 0.05* *P < 0.01* *-*
[30]-1	EA	6	GV20 GV29	2 Hz,1 mA, 20 min	CUMS only No intervention	6	Days 1–21	Gal Protein Gal mRNA	The 22nd day	Increased	*p*< 0.05 *p*< 0.01
[25]-1	EA	10	GV20 EX-HN3	2 Hz,1 mA, 20 min	Sham EA (GV20, EX-HN 3 no stimulation)	10	Days 33–59	TPH 5-HT1A	After behavioral tests	Increased	*p*< 0.01
[22]-1	EA	7	GV20 EX-HN 3	2 Hz, < 3 mA 0.2 ms,15 min	WKY only No intervention	6	Days 1–21	LTP GluN2B	After behavioral tests	Increased	*p*< 0.01
[35]-1	EA	10	GV20 GV29	1 mA, 2 Hz, 5 Hz, 30 min	CUMS only No intervention	10	Days 21–28	Body weight SPT OFT	After intervention termination	Increased	*p< 0.05*
[35]-2	EA + SSRI paroxetine (1.8 mg/kg)	15	GV20 GV29	1 mA, 2 Hz, 5 Hz, 30 min	EA + saline (1.8 mg/kg)	15	Days 21–28	pyramidal -cell PSD Mitochondrion	After behavioral tests	Increased	*-*
[40]-1	EA	10	GV20 GV29	2 Hz,1 mA, 20 min	CRS only No intervention	10	Days 1–21	IL-1beta IL-6	After behavioral tests	Decreased	*p*< 0.05
[40]-1	EA	8	DU20 GB34	2/100 Hz 0.3 mA 30 min	CUMS only No intervention	8	Weeks 7–11	ASC caspase-1 P2 × 7R IL-1β, IL-6 Iba-1,IL-18 GFAP- mRNA	After behavioral tests	Decreased Increased	*p*< 0.001
[33]-1	EA	10	GV20 EX-HN 3 ST40 LR3	40 Hz 15 min	CUMS only No intervention Maprotiline (10 mg/kg)	10 10	Days 2–23	Copper, zinc	After EA treatment	Decreased Increased	*p*< 0.05
[33]-1	EA	8	GV20	15 Hz 1 mA, 20 min	CUMS only No intervention/ catgut embedding (CE)	8 8	Days 1–21	CORT ACTH	24 h after the last EA treatment	Decreased	*p*< 0.01
[36]-1	EA	10	GV20 PC6 SP6	25 min	CUMS only No intervention	10	Days 29–42	BDNF	After behavioral tests	Increased	*p*< 0.05
[46]-1	EA	3	GV14 GV20	100 Hz, 30 min,0.2 ms1,2,3 mA	Sham EA (GV14 GV20 no stimulation)	3	weeks 2–6	TrkB	After EA treatment	Increased	*-*
[31]-1	EA	8	LI4 LR3	2 and 20 Hz, 30 min.	CUMS only No intervention	8	weeks 3–6	cAMP AC PKA	After behavioral tests	Increased	*p*< 0.01
[28]-1	EA	8	GV20 GB34	2 /100 Hz , 0.3 mA, 30 min.	CUMS only No intervention	8	Days 15–28	p-ERK	After behavioral tests	Increased	*P* < 0.01
[39]-1	EA	7	GV20 EX-HN3	2 Hz, < 3 mA 0.3 ms,15 min	Sham EA (GV20, EX-HN 3 no stimulation)	7	Days 15–28	p-ERK1/2/ ERK p-P38/P38	After behavioral tests	Increased	*p*< 0.01
[32]-1	EA	24	GV20 GV29	2 Hz, 0.6 mA, 20 min	CUMS only No intervention	24	Days 1–21	p-ERK	After behavioral tests	Increased	*p*< 0.05
[32]-2	EA+ PD98059 0.1 mg/kg	8	GV20 GV29	2 Hz, 0.6 mA, 20 min	EA GV20 GV29 2 Hz, 0.6 mA, 20 min	8	Days 1–21	p-ERK	After behavioral tests	Decreased	*p*< 0.05
[41]-1	EA	15	GV20 GV29	2 Hz, 1 mA, 20 minutes	CRS only No intervention	15	Days 1–28	Prkc DAT Th Mapt	Day 28	Decreased Decreased Increased Increased	*p*< 0.05 *p* > 0.05 *p*< 0.05 *p* > 0.05
[37]-1	EA	15	GV20 EX-HN3	2 Hz,1 mA 20 min	CUMS only No intervention	15	Days 1–21	Vgf, Igf2, Tmp32, Loc500373, Hif1a, Folr1, Nmb, Rtn	After behavioral tests	Increased	*-*
[47]-1	EA	10–13	GV20 GV29	2 Hz, 2 mA, 15 min	MS only No intervention	10–13	Days 61–81	SPT OFT	Days 81–96	Increased Decreased	*p*< 0.05 *p*< 0.01
[47]-2	EA	8–10	GV20 GV29	2 Hz, 2 mA, 15 min	Sham EA (GV20 GV29 no stimulation)	8–10	Days 61–81	ACTH CORT	After behavioral tests	Decreased	*p*< 0.001 *p*< 0.05
[47]-3	EA	8–10	GV20 GV29	2 Hz, 2 mA, 15 min	Sham EA (GV20 GV29 no stimulation)	8–10	Days 61–81	Ucp3, Cplx3, Dbp, Cdh12 LOC102555-167, LOC10-2555866, Npepo, Syt6	After behavioral tests	Increased Decreased	*p*< 0.05

*FST* forced swim test,* OFT* open-field test *MWM* morris water maze test,* EZM* elevated zero maze,* CRH* corticotropin-releasing hormone,* CORT* corticosterone *ACTH* adrenocorticotropicACTH* mBDNF* mature brain-derived neurotrophic factor *proBDNF* brain-derived neurotrophic factor precursorproBDNF* TrkB* receptor tropomyosin-related kinase receptor B, *HR* heart rate,* BP* blood pressure,* Gal* galanin,* TPH* tryptophan hydroxylase,* 5-HT* serotonin,* cAMP* cyclic adenosine monophosphate,* AC* adenylyl cyclase,* PKA* protein kinase A,* ERK* extracellular signal-regulated kinase,* LA* Locomotor activity,* p-ERK* phosphorylated extracellular signal-regulated kinase,* LTP* long-term potentiation,* PSD* postsynapse density,* Prkc* protein kinase C,* Mapt* microtubule-associated protein Tau,* DAT* dopamine transporter,* Th* tyrosine hydroxylase

^a^Days after injection

**Fig. 2:**
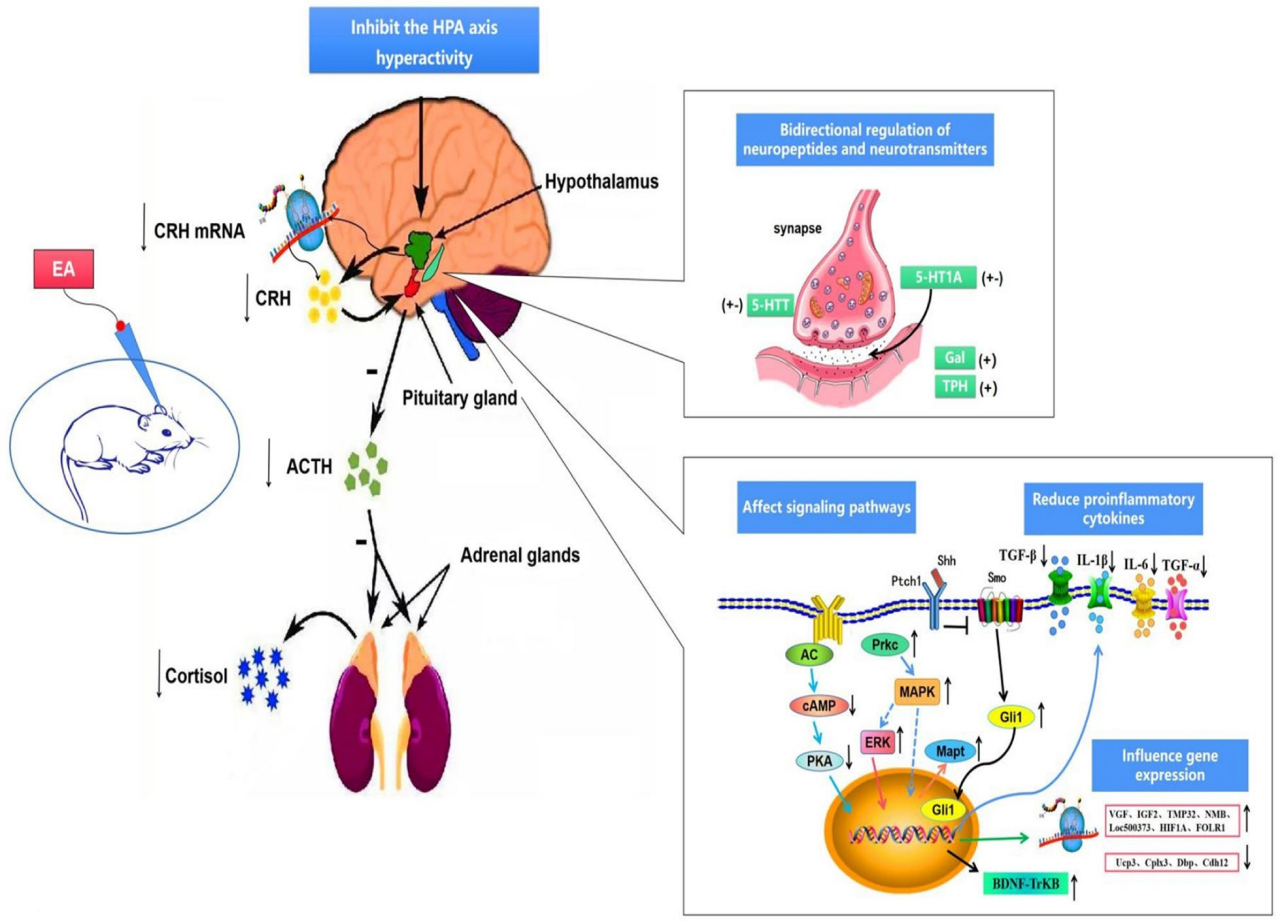
Mechanisms of EA for depression

Finally, we summarized the mechanism of EA in the treatment of depression (Fig. 2). There were 4 dimensions (synaptic plasticity dysfunction, the HPA axis, inflammation and gene expression) that required detailed analysis. One of the factors that causes depression is the hyperactivity of the HPA axis. EA can reduce corticotropin-releasing hormone (CRH) mRNA expression in the hypothalamus and the expression of CRH and adrenocorticotropic hormone (ACTH) in the hypothalamus and pituitary gland, thereby reducing cortisol expression in the adrenal glands and inhibiting the HPA axis. Monoamine neurotransmitters (serotonin, dopamine and noradrenaline) play an important role in depression and normal mood development [48]. EA can restore synaptic plasticity by regulating the expression of monoamine neurotransmitters in the hippocampus and then alleviate depression symptoms. Specifically, it can induce bidirectional regulation of the expression of serotonin transporters (5-HTTs) and serotonin-1A (5-HT1A), increase the expression of Gal and TPH in the hippocampus and restore synaptic plasticity. EA acts on 3 main signaling pathways to exert its effects in depression. First, it can downregulate the expression of cyclic adenosine monophosphate (cAMP) and protein kinase A (PKA) to affect the AC-cAMP-PKA signaling pathway. Second, EA can regulate the Ptch1-Shh-Smo pathway by downregulating the expression of the inflammatory factors IL-6, TGF-α, TGF-β and IL-1β. Finally, it upregulates the expression of protein kinase c (Prkc) and ERK to affect the MAPK pathway. EA also modulates depression by regulating the expression of some genes (Vgf, Igf2, Tmp32, Loc500373, Hif1a, Folr1, Ucp3, Cplx3, Dbp, and Cdh12). Generally, in rat models of depression, EA can inhibit excessive excitement of the HPA axis, reduce inflammatory cytokine levels, improve the expression of brain-derived neurotrophic factor (BDNF), restore hippocampal synaptic plasticity, induce bidirectional regulation of neuropeptides and neurotransmitters, and induce signal transduction pathways and genome expression to exert a beneficial effect in the treatment of depression.

## Discussion

To date, most animal studies on the therapeutic effect of EA on depression have been conducted in China. In most of these studies, the effect of EA on animal body weight was observed, and then a series of behavioral tests were carried to measure emotional and physical function. The FST is a common test of behavioral despair that is widely used to evaluate antidepressant-like effects in rodents [49]. The OFT is a commonly used test to qualitative and quantitatively measure the general locomotor activity and willingness to explore of rodents. This test is designed to measure anxiety and depression as well as behaviors such as locomotor activity and exploration [20]. The MWM test is used to evaluate spatial learning and memory [22]. The sucrose intake test is a behavioral task used to assess the degree of anhedonia in rats [39]. Furthermore, the mechanism of EA treatment in depression was further explored. In depression, EA exerts effects on neuropeptides and neurotransmitters; proinflammatory cytokines; serum copper, zinc, calcium and magnesium levels; the HPA axis; BDNF; signaling pathways; the genome; and hippocampal synaptic plasticity.

Most of the experiments used the GV29 (Yintang)/EX-HN3 (Yintang) and GV20 (Baihui) acupoints for EA treatment. Notably, a total of 19 studies used 2-Hz EA, and 5 studies used 100-Hz EA. There is evidence showing that 2- and 100-Hz stimulation may provide therapeutic effects through different mechanisms. The effect of 2-Hz EA is mostly related to the neuropeptide system, including corticotropin releasing factor, somatostatin, galanin, etc. [30, 38]. The function of 100-Hz EA may be related to the neurotrophic factor system, especially BDNF [36, 46]. In addition, it should be noted that the efficacy of EA at 2-100 Hz depends on the disease [50]. Wu et al. found that EA had analgesic effects at 2 Hz, 15 Hz and 100 Hz but that only 100-Hz EA significantly relieved depressive behavior [44]. Yang et al. compared the effects of 100-Hz (high-frequency) EA and 2-Hz (low-frequency) EA on depression [27]. They found that 2-Hz EA combined with low-dose citalopram significantly improved performance in the sucrose preference test and FST. Therefore, we speculate that when EA is selected as a single treatment, 100-Hz EA is more effective than 2-Hz EA in relieving negative symptoms. However, 2 Hz EA may be more suitable for combination therapy with low-dose antidepressants and may produce a greater antidepressant effect than single treatment.

The results of behavioral tests showed that the immobility time in the FST was significantly decreased and the distance of horizontal/vertical movements and number of entries into the center area in the OFT and the number of platform crossings and the surrounding area in the MWM were significantly increased in animals subjected to EA [20, 23, 24, 27, 29, 34, 34, 44]. In addition, other experiments revealed that EA treatment improved the body weight and sucrose preference index of depressed rats [24, 27, 34]. Of note, some studies have also pointed out that the combination of EA and a low dose of an antidepressant (citalopram/chloroimipramine) can produce greater therapeutic effects than either treatment alone [34, 42, 43]. Moreover, another study indicated that 100 Hz and the ST36 (Zusanli) SP6 (Sanyinjiao) acupoints may be the optimal parameters for improving physical pain and attenuating depressive-like behavior [44]. Importantly, EA at the auricular concha region (EA-ACR) has been shown to decrease the depressed rats’ mean heart rate and mean blood pressure [29]. Moreover, Zhou et al. [33] indicated that EA can regulate copper and zinc levels in depressed rats, exerting a potentially similar antidepressant effect as maprotiline.

Increasing evidence shows that the biological mechanism of depression involves synaptic plasticity, especially in the prefrontal cortex and hippocampus [51]. According to relevant research, the 5-HT system plays an important role in the treatment of depression [52]. Duan et al. [25] postulated that the antidepressant efficacy of EA treatment is achieved by enhancement of 5-HT synthesis, upregulation of 5-HT1A levels, and increasing 5-HT content in the brain and synaptic gaps. Additionally, Han et al. indicated that EA may alleviate depression-like behaviors and reverse the impairment in cornu ammonis 1 (CA1) synaptic plasticity in the hippocampus by downregulating the expression of 5-HTT and 5-HT1A receptors in depressed rats [21]. This research illustrates that EA can restore hippocampal synaptic plasticity by regulating 5-HT receptor levels. Hippocampal synaptic plasticity is responsible for learning and memory. Persistent changes in synaptic efficacy, long-term potentiation (LTP) and depression are considered cellular substrates underlying learning and memory processes [53]. She et al. [22] reported that tetanic stimulation failed to induce LTP and that the expression of NMDA receptor 2B (GluN2B) protein was significantly decreased in depressed rats, while EA reversed the impairment of LTP and restored GluN2B protein expression to normal levels. These data indicate that EA can enhance the expression of GluN2B to promote LTP and alleviate depression-like behavior. Zhang et al. observed a significant loss of PSD and damage to the mitochondrial ultrastructure and pyramidal cell layer in the hippocampus in CUMS rats. After EA intervention, the hippocampal pyramidal cell layer was restored, PSD increased and mitochondria were repaired [35]. These findings suggest that EA enhances antidepressant effects in depressed rats by protecting synaptic and mitochondrial functions in the hippocampus. These findings also indicate that the mechanism of SSRIs might involve alterations in hippocampal synaptic plasticity.

Moreover, mounting evidence suggests that proinflammatory cytokines play an important role in neurogenesis and neuroprotection. Chronic neuroinflammation due to the release of proinflammatory cytokines contributes to depression [54]. Psychological and physical stressors can activate immune and inflammatory processes, contributing to depressive symptoms [55]. Proinflammatory cytokines and some components of the inflammasome in the hippocampus have been implicated in the pathophysiology of depression [56, 57]. Guo et al. [40] reported that EA can downregulate the levels of interleukin-6 (IL-6) and IL-1beta in the hippocampi of depressed rats, demonstrating that the proinflammatory cytokines IL-1beta, IL-6, and TGF-beta mediate the onset of depressive symptoms and further suggesting that EA can potentially alleviate depression through a mechanism involving immunological modulation. Yue et al. [26] indicated that EA treatment can significantly decrease neuroinflammation in the hippocampi of depressed rats, such as by decreasing the expression of NLRP3 inflammasome components (ASC and caspase-1) and activating microglia and ATP-gated transmembrane (P2 × 7) receptor, Iba-1, IL-18, IL-1β, TNFα and IL-6 expression. The researchers speculated that EA can reverse depression-induced IL-1β-related microglial activation, which may be mediated by P2 × 7-NLRP3 inflammatory signaling.

The HPA axis is a core neuroendocrine signaling system involved in physiological homeostasis and the stress response. The HPA axis has been implicated as one of the key factors in the pathogenesis of depression. Consequently, HPA axis dysregulation is generally regarded as the diagnostic criterion in the early stages of depression [58]. Chen et al. [38] reported that EA markedly reduced serum corticosterone (CORT) and ACTH levels. In addition, Le et al. [34] reported that EA treatment decreased hypothalamic CRH mRNA expression as well as ACTH and CORT levels in the plasma. Many researchers regard neuropeptide systems (corticotropin-releasing factor, neuropeptide Y, galanin, vasopressin, substance p, somatostatin, etc.) as modulators of the behavioral states observed in mood disorders such as depression [59]. Based on this theory, Mo et al. [30] found that EA treatment upregulated the levels of hippocampal galanin in depressed rats to produce potential antidepressant effects. These data show that EA treatment can inhibit HPA axis hyperactivity, a potentially key mechanism in the etiology of antidepression. Consistent with this conclusion, a recent study revealed that ACTH and CORT levels in the plasma were higher in a rat model of maternal separation-induced depression than in healthy animals and that EA treatment reversed the increase in the concentration of ACTH and CORT in rats with maternal separation-induced depression [47].

As the most widely studied brain neurotrophin, BDNF promotes synaptic plasticity and neuronal growth and has been proposed as a biological marker of brain neuroplasticity; BDNF plays a key role in memory and cognition [60]. Some reports suggest that conventional antidepressants mediate antidepressant-like efficacy by upregulating BDNF expression in specific regions. Meta-analyses have shown that BDNF concentrations are lower in patients with depression than in healthy controls and that levels increase with successful antidepressant treatment [61]. In conclusion, BDNF is an essential determinant of antidepressant efficacy [62]. Consistent with the above theory, experiments have shown that EA can increase the expression of BDNF in the sera and hippocampi of depressed rats [27, 36]. Furthermore, related investigations have revealed that EA might modulate the BDNF-TrkB (tropomyosin-related kinase receptor B) interaction in the mesolimbic dopaminergic pathway to alleviate depressive-like symptoms [46]. All these data show that EA can prevent depression-induced decreases in BDNF signaling.

There is strong evidence that chronic stress leads to abnormal expression of stress-induced genes and that abnormal gene expression in turn worsens psychological disorders, resulting in a vicious cycle that affects the treatment of depression [63–67]. A previous study revealed multiple gene imbalances in the brains of depressive patients and speculated that polygenic disorders in the brain are a pathological factor of depression [68]. The use of rat genomic gene chip technology to evaluate the mechanism of EA could illuminate the multichannel, multitargeted and integrated regulatory effects of EA [69]. Duan et al. [37] profiled hippocampal gene expression changes in rats after EA therapy using gene chips and found that in the depression model group, the expression of genes related to inflammation/immunity and oxidative stress was increased, while the expression of genes related to transcription/translation, neurotransmission/signal transduction, metabolism, enzymatic reactions, metabolism and protein biosynthesis was decreased. These changes resulted in hippocampal structural and functional damage followed by the onset of depression symptoms. However, after EA treatment, the expression of these genes tended to return to normal. This study examined 8 genes (Vgf, Igf2, Tmp32, Loc500373, Hif1a, Folr1, Nmb, and Rtn) by RT-PCR, and the results indicated that EA modulated depression by regulating the expression of these particular genes. The researchers used microarray analysis and RNA sequencing and found that EA can alter mRNA and miRNA expression in depression rat models [27, 37]. Zheng et al. [47] used genome-wide RNA sequencing to explore the altered gene sets involved in circadian rhythm and neurotransmitter transporter activity in depressed rats and found that the expression of these gene sets tended to be reversed after EA treatment. The researchers found that downregulation of the expression of protective genes (Ucp3, Cplx3, Dbp and Cdh12) was reversed by EA treatment. Conversely, upregulation of the expression of damaging genes (LOC102555-167, LOC10-2555866, Npepo and Syt6) was reversed by EA treatment. These 2 studies suggest that EA ameliorates depression-related manifestations by regulating the expression of multiple genes.

The influence of EA on signaling pathways can be effectively studied on the basis of the modern theory of cell signaling. This theory also provides new ideas for clinical treatment from the molecular perspective. Most studies on the effect of EA on depression have focused on individual indicators of the chronic stress-related HPA axis, inflammation, serotoninergic system or dopaminergic system. Few studies have assessed the overall regulatory effect of EA on depression. Pathway analysis could reveal the most important biochemical metabolic pathways and signal transduction pathways associated with differentially expressed proteins. To determine the mechanism of EA in depression, 6 studies assessed the levels of signaling pathway-related factors that were imbalanced during depression and were subsequently normalized after EA treatment. Cai et al. [45] found that EA effectively resisted the effects of oxidation by reducing malondialdehyde (MDA) levels and increasing glutathione (GSH) levels in PSD rats. EA also reduced the levels of inflammatory cytokines, including IL6, TNFα, and IL1β. Furthermore, the upregulation of 5-HT levels verified that EA had an antidepressant effect. Moreover, the significant upregulation of Shh, Gli1, Smo, and Ptch1 expression in the EA group suggested that EA activated the hippocampal Shh signaling pathway. This study suggests that EA can effectively relieve depressive-like behaviors by suppressing inflammation and oxidative stress via activation of the hippocampal Shh signaling pathway. Liu et al. [31] reported that EA reversed the decrease in the cAMP level as well as the change in the ratio of adenylyl cyclase (AC) to PKA in depression model rats as effectively as fluoxetine. The results showed that the AC-cAMP-PKA postreceptor signal transduction pathway might be crucial for the ability of EA to alleviate depression symptoms. Additionally, EA greatly increased the number of phosphorylated extracellular signal-regulated kinase (p-ERK)/Nestin double-positive cells, the p-ERK level, the p-ERK1/2 ratio, the ratio of p-ERK1/2 to ERK levels, the p-p38 level, the ratio of p-p38 to p38 levels, and ERK phosphorylation. The above experiment demonstrates that EA attenuates depression-like behaviors induced by stress in rats, in part by activating the ERK signaling pathway [28, 32, 39]. Yang et al. [41] reported that microtubule-associated proteins (Mapt) and Prkc are important for the MAPK signaling pathway. EA downregulated the expression levels of Prkc and upregulated the expression levels of Mapt in depressed rats. These findings indicate that EA can alleviate depression through modulating the MAPK signaling pathway. Dopamine transporter (DAT) and tyrosine hydroxylase (Th) are involved in dopaminergic transmission. Notably, western blot analysis showed that the expression of Th was tended to decrease and that the expression of DAT tended to increase in the depressed rats, although the differences were not significant; these changes were reversed by EA intervention. The researchers speculated that EA might increase Th expression and decrease DAT expression to induce the release of DA into the synaptic cleft, resulting in the activation of the reward system and gave positive reinforcement for antidepressant therapy.

The behavioral changes induced by acupuncture treatment in depressed animals have been evaluated by meta-analyses and systematic reviews [70]. This article provides an update on the literature (through December 2019), adds the findings of research from recent years, and provides a comprehensive systematic review of the mechanism of EA in depression (as assessed by behavioral tests, molecular biology experiments, genomic analysis, etc.). Based on the strong evidence discussed in this review, we believe that EA exerts its antidepressant effect by regulating a variety of neurotransmitters to promote cholinergic nerve transmission, activate catecholamine and 5-hydroxytryptaminergic synaptic transmission, reshape neural synapses, simultaneously regulate a variety of neuropeptides (approximately 20–30) to activate the AC-cAMP-PKA cascade within the central nervous system, further enhance neurotrophic protein signal transduction, inhibit oxidative stress, inhibit the inflammatory response, and thus regulate the overall emotional state [71, 72]. We expect that this review will provide useful prospective theoretical bases for clinical acupuncture treatment. The study has some limitations. First, the mechanisms of rapid and chronic antidepressants might be different. Relevant clinical trials have reported that the efficacy, response rates and onset times of EA and antidepressants are different [17]. It can be inferred that there are differences in the mechanisms of EA and antidepressants. We believe that a possible mechanism underlying the rapid action of EA is stimulation of the brain through promotion of synaptic function or neuronal activity in the brain. There have been few studies on the effects of EA on depression, and most basic experiments have only compared the therapeutic effects of EA and antidepressants. Therefore, this article mostly focused on the mechanism of EA in the treatment of depression. However, with the increasing acceptance of EA, research on the different mechanisms of EA and antidepressants in depression will be very valuable. Future research should not only focus on the therapeutic effect of EA in depression but also the mechanistic differences between EA and antidepressants.

## Prospects for the use of EA of depression

Depression is a complex mental disorder that involves various brain nuclei, multiregional neuronal associations and diverse transmitters. However, the precise pathogenesis of depression is still unknown, and studies attempting to elucidate the mechanism of EA treatment for depression are still insufficient.

### P11 and the LHb

The habenula is involved in multiple processes that are affected in major depressive syndrome, such as reward processing, cognition, stress adaptation, sleep and circadian rhythm regulation and biological rhythms [73–75]. Studies suggest that the habenula plays a major role in the long-term modification of monoamine transmission and behavioral responses to stress and that dysfunction of the lateral habenula (LHb) is associated with psychiatric disorders, including major depression [76]. Based on these experiments [77–79], we can speculate that the LHb interacts with the 5-HT system, modulates *N*-methyl-D-aspartic acid (NMDA) and aminomethyl phosphonic acid (AMPA) receptors, and has a bidirectional connection with the HPA axis to regulate the stress-related adaptive response to depression. Thus, the LHb is a potential target for EA intervention in depression. We hope that future studies will elucidate the mechanism of EA in depression, specifically in relation to the habenula.

Furthermore, studies have revealed that p11 levels in the LHb are decreased in depressed suicide patients [80]. P11 (S100A10) is a multifunctional protein that interacts with serotonin receptors; therefore, p11 in the LHb is an important molecular determinant regulating depression. Chronic stress precipitates increased neuronal activity in the LHb, leading to a decrease in dopaminergic and serotonergic neurotransmission and inducing depressive symptomatology, all of which are inhibited in the presence p11 [77]. The LHb is a brain region that is crucial for regulation and resilience in depressed patients, but the molecular determinants responsible for the key role of the LHb in depression remain elusive. Therefore, future research could focus on the effect of EA intervention on p11 in the LHb, which may help elucidate the molecular and cellular basis of depression.

### The CA1, CA2, CA3, DG regions

Many 5-HT receptors are associated with depression and vary across brain regions [21]. In one study, qRT-PCR was used to evaluate the expression of 5-HT receptors and P11 protein in the prefrontal cortex, hippocampus, amygdala and raphe nucleus in depressed suicidal patients. According to this study, differences in 5-HT1A, 5-HT1B and p11 mRNA expression between the frontopolar cortex and hippocampus were relatively widespread and were dependent on sex [80]. The hippocampus, as an important brain region in the limbic system, plays a key role in cognitive function and emotional regulation. The hippocampus is composed of the hippocampal gyrus and dentate gyrus (DG). The hippocampal gyrus includes the CA1-CA4 regions, which are mainly composed of pyramidal neurons. The DG region is mainly composed of granule neurons. Clinical studies have reported that the volume of the CA1-CA3 and DG regions in the hippocampi is smaller in depressed patients than in the unaffected population [81]. A high level of spontaneous apoptosis in the CA1 and DG regions has been found in patients with major depression [82]. In addition, the number of new neurons and the volume of the granular cell layer in the DG region in depression model rats are significantly reduced, as is the number of neurons in the CA3 region  [83]. Researchers have reported that the antidepressant effects of fluoxetine are mediated by increased neurogenesis in the adult DG region [84]. The DG region is an important brain region for the regeneration of new neurons. At present, the absence of new neurons is considered one of the key factors in the pathogenesis and treatment of depression [85, 86]. It has been confirmed that changes in hippocampal structure and function are related to depression. However, the specific part of the hippocampus that is associated with depression remains unclear. In recent years, a large amount of research has focused on the CA1 region, while the CA2, CA3 and DG regions have been less studied. Therefore, we suggest that future studies on acupuncture-based treatment of depression should explore in detail the specific functions of hippocampal brain regions (including the CA1–CA4 and DG regions).

### Gut‐brain axis

Based on the pathogenesis of depression, depression is not only a brain disorder but is also closely related to the function of various parts of the body, especially the endocrine system and immune system  [87]. Modern lifestyles have become the basis of immune system dysfunction, and disorders of gut microbiome composition can lead to depression [88]. In recent years, studies have revealed a strong bidirectional relationship between the gut and brain. Changes to the gut microflora strongly affect psychological function. The levels of lactobacilli, bifidobacteria, Firmicutes, Faecalibacterium and Ruminococcus are decreased in depressed patients compared with healthy individuals, while the levels of Protobacteria, Bacteroides, and Proteobacteria are increased [89, 90]. This suggests that the microbiomes of depressed patients exhibit low-diversity dysbiosis. To test whether depressed patients have a specific gut microbiota composition and to determine the impact of such a microbiotic composition, the feces (depressed microbiota) of depressed patients were transplanted into germ-free mice. The experimental animals were found to have depressive symptoms, and it was speculated that gut microbiota dysbiosis might be one underlying factor of depression  [91]. According to traditional Chinese medicine, acupuncture can regulate the whole body, even when the treatment is applied to the head. Acupuncture can bidirectionally regulate the brain and gut, which is consistent with the modern concept of the gut-brain axis. Therefore, further studies should focus on the impact of EA intervention on the gut microbiota in depression model rats and explore whether the bidirectional regulatory effect of EA on the gut-brain axis can be harnessed to alleviate depression.

### Neural network activity

Some large-scale brain networks, including default mode networks, frontal parietal and dorsal attention networks, and salience networks, are considered potential neural substrates of depression [92, 93]. A study on depressed patients with anxiety revealed increased resting-state activity in the anterior insula, and these results suggest that alterations in cortico-limbic networks may play a critical role in the pathogenesis of depression [94]. Another study revealed that an increase in intrinsic neural oscillations in the right bilateral dorsolateral prefrontal cortex in the resting state is a characteristic change in the depressive state and merits further investigation as a potential imaging marker for depression [95]. Additionally, clinical studies have reported that EA has a faster onset of action than other treatments in depression and can modulate abnormal aberrant amygdala networks in depressed patients [14, 17]. Transcutaneous auricular vagus nerve stimulation can significantly increase amygdala-dorsolateral prefrontal cortex connectivity, which is associated with the degree of depression [96]. These results suggest that the antidepressant effect of EA might be related to the inhibition of abnormal discharges from the LHb and the regulation of the reward system. These studies indicate that the regulatory effect of EA on the limbic-paralimbic-neocortical network is also an important mechanism for the treatment of depression [97]. With the advancement of functional magnetic resonance imaging (fMRI), further research on the regulatory effect of EA on neural network activity in depression may provide important information.

## Conclusions

Research on the therapeutic effect of EA in depression has progressed considerably in recent years. It has been established that EA treatment involves multiple therapeutic mechanisms, including inhibition of HPA axis hyperactivity, increased expression of BDNF, regulation of neuropeptides and neurotransmitters, promotion of signaling pathways, modulation of the expression of particular genes, reductions in proinflammatory cytokine levels and restoration of hippocampal synaptic plasticity. However, a comprehensive theory of the mechanism by which EA can treat depression has not yet been proposed. Therefore, it is imperative that future researchers work to identify innovative scientific principles to explain the effectiveness of EA in depression. Depression is related not only to the brain but also to the overall function of the human body. Previous studies have not identified the specific brain regions involved in depression or the relationship between these regions and the response of the body. We suggest that EA may exert a therapeutic effect in depression by acting as a trigger for network regulation in the brain. This implies that the regulatory function of EA is holistic in nature. Additional research on the mechanism of EA in depression could focus on the characteristics of EA from a multiangle and multitarget perspective. We await further results to present useful prospective theoretical bases for clinical acupuncture treatment.

## Data Availability

All data used in this systematic review are fully available in the public domain.

## Abbreviations

AC: Adenylyl cyclase
ACTH: Adrenocorticotropic hormone
AMPA: Aminomethyl phosphonic acid
BDNF: Brain-derived neurotrophic factor
cAMP: Cyclic adenosine monophosphate
CNKI: Chinese National knowledge Infrastructure
CORT: Corticosterone
CUMS: Chronic unpredictable mild stress
CRS: Chronic restraint stress
CMS: Chronic mild stress
CA1: Cornu ammonis 1
DG: Dentate gyrus
DAT: Dopamine transporter
EA: Electroacupuncture
EA-ACR: EA at the auricular concha region
ERK: Extracellular regulated protein kinases
MAPK: Mitogen-activated protein kinase
MDA: Malondialdehyde
Mapt: Microtubule-associated proteins
PSD: Poststroke depression
PKA: Protein kinase A
Prkc: Protein kinase c
P2 × 7: ATP-gated transmembran
MFB: Medial forebrain bundle
WKY: Wistar Kyoto
WHO: World Health Organization
WOS: Web of Science
FST: Forced swim test
fMRI: Functional magnetic resonance imaging
OFT: Open-field test
MWM: Morris water maze test
NMDA: *N*-methyl-D-aspartic acid
GluN2B: NMDA receptor 2B
Gal: Galanin
GSH: Glutathione
SPT: Sucrose-preference test
SSRIs: Selective serotonin reuptake inhibitors
TPH: Tryptophan hydroxylase
Th: Tyrosine hydroxylase
HPA: Hypothalamic-pituitary-adrenal
IL-6: Interleukin-6
5-HT: Serotonin
5-HTTs: Serotonin transporters
5-HT1A: Serotonin-1A
LTP: Long-term potentiation
LHb: Lateral habenula

## References

[1] Trimmer PC, Higginson AD, Fawcett TW, McNamara JM, Houston AI, Evolution (2015). Adaptive learning can result in a failure to profit from good conditions: implications for understanding depression. Med Public Health.

[2] Dennis CL, Dowswell T (2013). Interventions (other than pharmacological, psychosocial or psychological) for treating antenatal depression. Cochrane Database Syst Rev..

[3] Black CN, Bot M, Scheffer PG, Cuijpers P, Penninx BW (2015). Is depression associated with increased oxidative stress? A systematic review and meta-analysis. Psychoneuroendocrinology.

[4] Meng X, Brunet A, Turecki G, Liu A, D’Arcy C, Caron J (2017). Risk factor modifications and depression incidence: a 4-year longitudinal Canadian cohort of the Montreal Catchment Area Study. BMJ Open.

[5] Rihmer Z, Gonda X (2011). Antidepressant-resistant depression and antidepressant-associated suicidal behaviour: the role of underlying bipolarity. Depress Res Treat.

[6] Fabbri C, Marsano A, Balestri M, De Ronchi D, Serretti A (2013). Clinical features and drug induced side effects in early versus late antidepressant responders. J Psychiatric Res.

[7] Taylor MJ, Rudkin L, Bullemor-Day P, Lubin J, Chukwujekwu C, Hawton K (2013). Strategies for managing sexual dysfunction induced by antidepressant medication. Cochrane Database Syst Rev.

[8] Harada T, Inada K, Yamada K, Sakamoto K, Ishigooka J (2014). A prospective naturalistic study of antidepressant-induced jitteriness/anxiety syndrome. Neuropsychiatr Dis Treat.

[9] Zhang J, Chen J, Chen J (2014). Early filiform needle acupuncture for poststroke depression: a meta-analysis of 17 randomized controlled clinical trials. Neural Regen Res.

[10] Lu L, Zhang XG, Zhong LL (2016). Acupuncture for neurogenesis in experimental ischemic stroke: a systematic review and meta-analysis. Sci Rep.

[11] Shin HK, Lee SW, Choi BT (2017). Modulation of neurogenesis via neurotrophic factors in acupuncture treatments for neurological diseases. Biochem&nbsp;Pharmacol.

[12] Yang JW, Li QQ, Li F, Fu QN, Zeng XH, Liu CZ (2014). The holistic effects of acupuncture treatment. Evid Based Complement Alternat Med.

[13] Kim YD, Heo I, Shin BC, Crawford C, Kang HW, Lim JH (2013). Acupuncture for posttraumatic stress disorder: a systematic review of randomized controlled trials and prospective clinical trials. Evid Based Complement Alternat Med.

[14] Duan G, He Q, Pang Y (2019). Altered amygdala resting-state functional connectivity following acupuncture stimulation at BaiHui (GV20) in first-episode drug-Naive major depressive disorder. Brain Imaging Behav.

[15] Li Q, Yue N, Liu SB (2014). Effects of chronic electroacupuncture on depression- and anxiety-like behaviors in rats with chronic neuropathic pain. Evid Based Complement Alternat Med.

[16] Yang X, Gong W, Ma X (2019). Factor analysis of electroacupuncture and selective serotonin reuptake inhibitors for major depressive disorder: an 8-week controlled clinical trial. Acupunct Med.

[17] Sun H, Zhao H, Ma C (2013). Effects of electroacupuncture on depression and the production of glial cell line-derived neurotrophic factor compared with fluoxetine: a randomized controlled pilot study. J Altern Complement Med.

[18] Banasr M, Valentine GW, Li XY, Gourley SL, Taylor JR, Duman RS (2007). Chronic unpredictable stress decreases cell proliferation in the cerebral cortex of the adult rat. Biol Psychiatry.

[19] DaSilva JK, Husain E, Lei Y, Mann GL, Tejani-Butt S, Morrison AR (2011). Social partnering significantly reduced rapid eye movement sleep fragmentation in fear-conditioned, stress-sensitive Wistar-Kyoto rats. Neuroscience.

[20] Xu S, Li S, Shen X, Meng X, Lao L (2011). Effects of electroacupuncture on depression in a rat model. Acupunct Electrother Res.

[21] Han X, Wu H, Yin P (2018). Electroacupuncture restores hippocampal synaptic plasticity via modulation of 5-HT receptors in a rat model of depression. Brain Res Bull.

[22] She Y, Xu J, Duan Y (2015). Possible antidepressant effects and mechanism of electroacupuncture in behaviors and hippocampal synaptic plasticity in a depression rat model. Brain Res.

[23] Guo Z, Tu Y, Guo TW (2015). Electroacupuncture pretreatment exhibits anti-depressive effects by regulating hippocampal proteomics in rats with chronic restraint stress. Neural Regen Res.

[24] Duan DM, Dong X, Tu Y, Liu P (2016). A microarray study of chronic unpredictable mild stress rat blood serum with electro-acupuncture intervention. Neurosci&nbsp;Lett.

[25] Duan D, Tu Y, Yang X, Liu P (2016). Electroacupuncture restores 5-HT system deficit in chronic mild stress-induced depressed rats. Evid Based Complement Alternat Med.

[26] Yue N, Li B, Yang L (2018). Electro-acupuncture alleviates chronic unpredictable stress-induced depressive- and anxiety-like behavior and hippocampal neuroinflammation in rat model of depression. Front Mol Neurosci.

[27] Yang J, Pei Y, Pan YL (2013). Enhanced antidepressant-like effects of electroacupuncture combined with citalopram in a rat model of depression. Evid Based Complement Alternat Med.

[28] Yang L, Yue N, Zhu X (2013). Electroacupuncture upregulates ERK signaling pathways and promotes adult hippocampal neural progenitors proliferation in a rat model of depression. BMC Complement Altern Med.

[29] Liu RP, Fang JL, Rong PJ (2013). Effects of electroacupuncture at auricular concha region on the depressive status of unpredictable chronic mild stress rat models. Evid Based Complement Alternat Med.

[30] Mo Y, Yao H, Song H (2014). Alteration of behavioral changes and hippocampus galanin expression in chronic unpredictable mild stress-induced depression rats and effect of electroacupuncture treatment. Evid Based Complement Alternat Med.

[31] Liu JH, Wu ZF, Sun J, Jiang L, Jiang S, Fu WB (2012). Role of AC-cAMP-PKA cascade in antidepressant action of electroacupuncture treatment in rats. Evid Based Complement Alternat Med.

[32] Li W, Zhu Y, Saud SM (2017). Electroacupuncture relieves depression-like symptoms in rats exposed to chronic unpredictable mild stress by activating ERK signaling pathway. Neurosci Lett.

[33] Zhou HH, Lu F, Chen SD, Zhou ZH, Han YZ, Hu JY (2011). Effect of electroacupuncture on serum copper, zinc, calcium and magnesium levels in the depression rats. J Tradit Chin Med.

[34] Le JJ, Yi T, Qi L, Li J, Shao L, Dong JC (2016). Electroacupuncture regulate hypothalamic-pituitary-adrenal axis and enhance hippocampal serotonin system in a rat model of depression. Neurosci Lett.

[35] Zhang J, Zhang Z, Zhang J (2019). Electroacupuncture improves antidepressant effects in cums rats by protecting hippocampal synapse and mitochondrion: an ultrastructural and iTRAQ Proteomic Study. Evid Based Complement Alternat Med.

[36] Mu D, Huang X (2015). Anti-depression effects of electroacupuncture through up-regulating serum E 2 and BDNF and expression of BDNF in hippocampus in chronic depression rats. J Acupunct Tuina Sci.

[37] Duan D, Yang X, Ya T, Chen L (2014). Hippocampal gene expression in a rat model of depression after electroacupuncture at the Baihui and Yintang acupoints. Neural Regen Res.

[38] Chen HD, Jin LQ, Ran L, Zhang LM (2012). Effects of electroacupuncture on rat model of chronic stress-induced depression. J Acupunct Tuina Sci.

[39] Xu J, She Y, Su N, Zhang R, Lao L, Xu S (2015). Effects of electroacupuncture on chronic unpredictable mild stress rats depression-like behavior and expression of p-ERK/ERK and p-P38/P38. Evid Based Complement Alternat Med.

[40] Guo T, Guo Z, Yang X (2014). The alterations of IL-1Beta, IL-6, and TGF-beta levels in hippocampal CA3 region of chronic restraint stress rats after electroacupuncture (EA) pretreatment. Evid Based Complement Alternat Med.

[41] Yang X, Guo Z, Lu J (2017). The Role of MAPK and dopaminergic synapse signaling pathways in antidepressant effect of electroacupuncture pretreatment in chronic restraint stress rats. Evid Based Complement Alternat Med.

[42] Yu J, Li XY, Cao XD, Wu GC (2006). Sucrose preference is restored by electro-acupuncture combined with chlorimipramine in the depression-model rats. Acupunct Electrother Res.

[43] Yu J, Liu Q, Wang YQ (2007). Electroacupuncture combined with clomipramine enhances antidepressant effect in rodents. Neurosci Lett.

[44] Wu YY, Jiang YL, He XF (2015). Effects of electroacupuncture with dominant frequency at SP 6 and ST 36 based on meridian theory on pain-depression dyad in rats. Evid Based Complement Alternat Med.

[45] Cai W, Ma W, Wang GT, Li YJ, Shen WD (2019). Antidepressant, anti-inflammatory, and antioxidant effects of electroacupuncture through sonic hedgehog-signaling pathway in a rat model of poststroke depression. Neuropsychiatr Dis Treat.

[46] Sun M, Wang K, Yu Y (2016). Electroacupuncture alleviates depressive-like symptoms and modulates BDNF signaling in 6-hydroxydopamine rats. Evid Based Complement Alternat Med.

[47] Zheng Y, He J, Guo L (2019). Transcriptome analysis on maternal separation rats with depression-related manifestations ameliorated by electroacupuncture. Front Neurosci.

[48] Evrensel A, Unsalver BO, Ceylan ME (2019). Neuroinflammation. Gut-brain axis and depression. Psychiatry Investig.

[49] Yang L, Yue N, Zhu X (2014). Electroacupuncture promotes proliferation of amplifying neural progenitors and preserves quiescent neural progenitors from apoptosis to alleviate depressive-like and anxiety-like behaviours. Evid Based Complement Alternat Med.

[50] Han JS (2011). Acupuncture analgesia: areas of consensus and controversy. PAIN.

[51] Bannerman DM, Sprengel R, Sanderson DJ (2014). Hippocampal synaptic plasticity, spatial memory and anxiety. Nat Rev Neurosci.

[52] Mouri A, Ikeda M, Koseki T, Iwata N, Nabeshima T (2016). The ubiquitination of serotonin transporter in lymphoblasts derived from fluvoxamine-resistant depression patients. Neurosci Lett.

[53] Muzio L, Brambilla V, Calcaterra L, D’Adamo P, Martino G, Benedetti F (2016). Increased neuroplasticity and hippocampal microglia activation in a mice model of rapid antidepressant treatment. Behav Brain Res.

[54] Kim YK, Na KS, Myint AM, Leonard BE (2016). The role of pro-inflammatory cytokines in neuroinflammation, neurogenesis and the neuroendocrine system in major depression. Prog Neuropsychopharmacol Biol Psychiatry.

[55] Iwata M, Ota KT, Duman RS (2013). The inflammasome: pathways linking psychological stress, depression, and systemic illnesses. Brain Behav Immunity.

[56] Xu Y, Sheng H, Bao Q, Wang Y, Lu J, Ni X (2016). NLRP3 inflammasome activation mediates estrogen deficiency-induced depression- and anxiety-like behavior and hippocampal inflammation in mice. Brain Behav Immunity.

[57] Zhang Y, Liu L, Peng YL (2014). Involvement of inflammasome activation in lipopolysaccharide-induced mice depressive-like behaviors. CNS Neurosci Ther.

[58] Du X, Pang TY (2015). Is dysregulation of the HPA-Axis a core pathophysiology mediating co-morbid depression in neurodegenerative diseases?. Front Psychiatry.

[59] Rotzinger S, Lovejoy DA, Tan LA (2010). Behavioral effects of neuropeptides in rodent models of depression and anxiety. Peptides.

[60] Mosqueiro BP, Fleck MP, Da RN (2019). Increased levels of brain-derived neurotrophic factor are associated with high intrinsic religiosity among depressed inpatients. Front Psychiatry.

[61] Kishi T, Yoshimura R, Ikuta T, Iwata N (2017). Brain-derived neurotrophic factor and major depressive disorder: evidence from meta-analyses. Front Psychiatry.

[62] Bjorkholm C, Monteggia LM (2016). BDNF—a key transducer of antidepressant effects. Neuropharmacology.

[63] Revollo HW, Qureshi A, Collazos F, Valero S, Casas M (2011). Acculturative stress as a risk factor of depression and anxiety in the Latin American immigrant population. Int Rev Psychiatry.

[64] Rogoz Z, Skuza G, Legutko B (2005). Repeated treatment with mirtazepine induces brain-derived neurotrophic factor gene expression in rats. J Physiol Pharmacol.

[65] Kohli MA, Salyakina D, Pfennig A (2010). Association of genetic variants in the neurotrophic receptor-encoding gene NTRK2 and a lifetime history of suicide attempts in depressed patients. Arch Gen Psychiatry.

[66] Schosser A, Butler AW, Ising M (2011). Genomewide association scan of suicidal thoughts and behaviour in major depression. PLoS ONE.

[67] Perroud N, Uher R, Ng MY (2012). Genome-wide association study of increasing suicidal ideation during antidepressant treatment in the GENDEP project. Pharmacogenom J.

[68] Porter RJ, Mulder RT, Joyce PR, Miller AL, Kennedy M (2008). Tryptophan hydroxylase gene (TPH1) and peripheral tryptophan levels in depression. J Affect Disord.

[69] Mo YP, Yao HJ, Song HT, Xu AP, Tang YS, Li ZG (2014). Progress of animal research on electro-acupuncture treatment for depression(big up tri, open). Chin Med Sci J.

[70] Kou RZ, Chen H, Yu ML, Xu TC, Fu SP, Lu SF (2017). Acupuncture for behavioral changes of experimental depressive disorder: a systematic review and meta-analysis. Sci Rep.

[71] Leung MC, Yip KK, Ho YS, Siu FK, Li WC, Garner B (2014). Mechanisms underlying the effect of acupuncture on cognitive improvement: a systematic review of animal studies. J Neuroimmune Pharmacol.

[72] Smith CA, Armour M, Lee MS, Wang LQ, Hay PJ (2018). Acupuncture for depression. Cochrane Database Syst Rev.

[73] Boulos LJ, Darcq E, Kieffer BL (2017). Translating the habenula-from rodents to humans. Biol Psychiatry.

[74] Browne CA, Hammack R, Lucki I (2018). Dysregulation of the lateral habenula in major depressive disorder. Front Synaptic Neurosci.

[75] Mathis V, Cosquer B, Barbelivien A (2018). The lateral habenula interacts with the hypothalamo-pituitary adrenal axis response upon stressful cognitive demand in rats. Behav Brain Res.

[76] Proulx CD, Hikosaka O, Malinow R (2014). Reward processing by the lateral habenula in normal and depressive behaviors. Nat Neurosci.

[77] Seo JS, Zhong P, Liu A, Yan Z, Greengard P (2018). Elevation of p11 in lateral habenula mediates depression-like behavior. Mol Psychiatry.

[78] Gold PW, Kadriu B (2019). A major role for the lateral habenula in depressive illness: physiologic and molecular mechanisms. Front Psychiatry.

[79] Zhang J, Wang Y, Sun YN (2019). Blockade of calcium-permeable AMPA receptors in the lateral habenula produces increased antidepressant-like effects in unilateral 6-hydroxydopamine-lesioned rats compared to sham-lesioned rats. Neuropharmacology.

[80] Anisman H, Du L, Palkovits M (2008). Serotonin receptor subtype and p11 mRNA expression in stress-relevant brain regions of suicide and control subjects. J Psychiatry Neurosci.

[81] Huang Y, Coupland NJ, Lebel RM (2013). Structural changes in hippocampal subfields in major depressive disorder: a high-field magnetic resonance imaging study. Biol Psychiatry.

[82] Lucassen PJ, Muller MB, Holsboer F (2001). Hippocampal apoptosis in major depression is a minor event and absent from subareas at risk for glucocorticoid overexposure. Am J Pathol.

[83] Kempermann G, Kronenberg G (2003). Depressed new neurons–adult hippocampal neurogenesis and a cellular plasticity hypothesis of major depression. Biol Psychiatry.

[84] Santarelli L, Saxe M, Gross C (2003). Requirement of hippocampal neurogenesis for the behavioral effects of antidepressants. Science.

[85] David DJ, Wang J, Samuels BA (2010). Implications of the functional integration of adult-born hippocampal neurons in anxiety-depression disorders. Neuroscientist.

[86] Dias GP, Cavegn N, Nix A (2012). The role of dietary polyphenols on adult hippocampal neurogenesis: molecular mechanisms and behavioural effects on depression and anxiety. Oxid Med Cell Longev.

[87] Belmaker RH, Agam G (2008). Major depressive disorder. N Engl J Med.

[88] Evrensel A, Ceylan ME (2015). The gut-brain axis: the missing link in depression. Clin Psychopharmacol Neurosci.

[89] Aizawa E, Tsuji H, Asahara T (2016). Possible association of Bifidobacterium and Lactobacillus in the gut microbiota of patients with major depressive disorder. J Affect Disord.

[90] Liu Y, Zhang L, Wang X (2016). Similar fecal microbiota signatures in patients with diarrhea-predominant irritable bowel syndrome and patients with depression. Clin Gastroenterol Hepatol.

[91] Zheng P, Zeng B, Zhou C (2016). Gut microbiome remodeling induces depressive-like behaviors through a pathway mediated by the host’s metabolism. Mol Psychiatry.

[92] Kaiser RH, Andrews-Hanna JR, Wager TD, Pizzagalli DA (2015). Large-scale network dysfunction in major depressive disorder: a meta-analysis of resting-state functional connectivity. JAMA Psychiatry.

[93] Mulders PC, van Eijndhoven PF, Schene AH, Beckmann CF, Tendolkar I (2015). Resting-state functional connectivity in major depressive disorder: a review. Neurosci Biobehav Rev.

[94] Liu CH, Ma X, Song LP (2015). Abnormal spontaneous neural activity in the anterior insular and anterior cingulate cortices in anxious depression. Behav Brain Res.

[95] Liu CH, Guo J, Lu SL (2018). Increased salience network activity in patients with insomnia complaints in major depressive disorder. Front Psychiatry.

[96] Liu CH, Yang MH, Zhang GZ (2020). Neural networks and the anti-inflammatory effect of transcutaneous auricular vagus nerve stimulation in depression. J Neuroinflamm.

[97] Hui KK, Marina O, Liu J, Rosen BR, Kwong KK (2010). Acupuncture, the limbic system, and the anticorrelated networks of the brain. Auton Neurosci.

