# Correction to: The mechanism of electroacupuncture for depression on basic research: a systematic review

**DOI:** 10.1186/s13020-021-00430-5

**Published:** 2021-02-10

**Authors:** Xuke Han, Yang Gao, Xuan Yin, Zhangjin Zhang, Lixing Lao, Qiu Chen, Shifen Xu

**Affiliations:** 1 Hospital of Chengdu University of Traditional Chinese Medicine, Chengdu, 610072 Sichuan China; 2 Shanghai Municipal Hospital of Traditional Chinese Medicine, Shanghai University of Traditional Chinese Medicine, Shanghai, 200071 China; 3 School of Chinese Medicine, The University of Hong Kong, Pokfulam, Hong Kong SAR, China; 4 Virginia University of Integrative Medicine, Fairfax, VA 22031 USA

## Correction to: Chin Med (2021) 16:10 10.1186/s13020-020-00421-y

Following publication of the original article [1], the authors identified an error that the author Shifen Xu is not indicated as the corresponding author.

Qiu Chen and Shifen Xu should be co-corresponding authors for this article.

The author group has been updated above and the original article [1] has been corrected.
